# On modelling disordered crystal structures through restraints from molecule-in-cluster computations, and distinguishing static and dynamic disorder

**DOI:** 10.1107/S2052252521000531

**Published:** 2021-02-18

**Authors:** Birger Dittrich

**Affiliations:** a Novartis Campus, Novartis Pharma AG, Postfach, Basel, CH-4002, Switzerland; b Mathematisch-Naturwissen­schaftliche Fakultät, Universität Zürich, Winterthurerstrasse 190, Zürich, CH-8057, Switzerland

**Keywords:** quantum crystallography, crystal structures, disorder refinement, molecule-in-cluster optimizations

## Abstract

Distinguishing disorder into static and dynamic based on multi-temperature X-ray or neutron diffraction experiments is the current state of the art, but is only descriptive, not predictive. Here, several disordered structures are revisited from the Cambridge Crystallographic Data Center ‘drug subset’, the Cambridge Structural Database and own earlier work, where experimental intensities of Bragg diffraction data were available. Using the molecule-in-cluster approach, structures with distinguishable conformations were optimized separately, as extracted from available or generated disorder models of the respective disordered crystal structures. Re-combining these ‘archetype structures’ by restraining positional and constraining displacement parameters for conventional least-squares refinement, based on the optimized geometries, then often achieves a superior fit to the experimental diffraction data compared with relying on experimental information alone. It also simplifies and standardizes disorder refinement. Ten example structures were analysed. It is observed that energy differences between separate disorder conformations are usually within a small energy window of *RT* (*T* = crystallization temperature). Further computations classify disorder into static or dynamic, using single experiments performed at one single temperature, and this was achieved for propionamide.

## Abstract 2

Restraints extracted from molecule-in-cluster optimizations of separate disorder components permit improved least-squares modelling of experimental crystal structures, and considering energy barriers alongside enthalpy differences further enables the distinguishing of disorder into static and dynamic.

## 1. Introduction   

More than 20% of experimentally determined crystal structures deposited in the Cambridge Structural Database (CSD) (Groom *et al.*, 2016 ▸) are disordered. Studying disorder is fascinating because many open questions remain. For the pharmaceutical industry, disorder is especially relevant in the context of crystal structure prediction (CSP) (Reilly *et al.*, 2016 ▸, and references therein). CSP is part of the emerging field of quantum crystallography (Genoni *et al.*, 2018 ▸), where the aim is to combine quantum chemical methods developed in the context of theoretical chemistry and apply them to research questions in solid-state physical chemistry. In the industry setting, these methods are applied to pharmaceutically relevant molecules. Taking disorder into account in the computational procedure can lead to energy re-ranking (Neumann *et al.*, 2015 ▸; Woollam *et al.*, 2018 ▸) in CSP energy landscapes, where the current paradigm for assessing polymorphism risk is that structures of high density and low enthalpy are most likely to crystallize (Price, 2013 ▸). Since energy ranking affects the risk assessment and the predictive value of CSP, I am here concerned with better modelling of disordered structures, and with distinguishing static and dynamic disorder.

An introduction to disorder in crystal structures can, for example, be found in the book by Müller *et al.* (2006 ▸). The authors note that since a crystal is a spatial average of a pattern (*e.g.* a molecule, an ion pair), disorder contradicts 3D periodicity. Furthermore, a distinction between substitutional and discrete (static) and continuous (dynamic) disorder is made. Substitutional disorder is introduced as being distinct from positional disorder in that different elements can be present in a structure at once, for example in minerals or salts. Positional disorder, where an atom occupies more than a single site, can be considered to include substitutional disorder.

To classify disorder into static and dynamic requires multi-temperature single-crystal X-ray diffraction (SC-XRD) experiments (Bürgi & Capelli, 2000 ▸) and is empirical. It is not usually attempted to classify the nature of disorder into static or dynamic based on a single SC-XRD experiment. Multi-temperature studies show that dynamic disorder is reduced at lower temperatures. For disorder to be present, the following conditions – here generalized beyond the case of azulene (Dittrich *et al.*, 2018 ▸) – need to be fulfilled. (i) A steric requirement: an overlay of idealized non-disordered molecular conformations in a disordered structure should exhibit a similar shape to each or both of the individual components. (ii) A Coulomb requirement: the overlay should not lead to considerably different charge distributions. (iii) A requirement concerning intra- and inter-molecular interactions: in all orientations, hydrogen bonds or other interactions should be of similar number and strength. A fourth requirement concerning energy will be added in this work.

It will also be reported how to improve the technical modelling procedure of disordered crystal structures by introducing restraints (Watkin, 1994 ▸) from molecule-in-cluster geometry optimizations (Dittrich *et al.*, 2020*a* ▸) of archetype structures (Dittrich *et al.*, 2020*b* ▸). Initial coordinates of archetype^1^ structures can be extracted from disordered experimental structures and then optimized. Re-combining optimized archetype structures, including all atoms (and not just the parts that are disordered), and constraining and/or restraining so-derived input for further least-squares (LSQ) refinement, then achieves a significantly superior fit to the experimental diffraction data compared with relying on experimental data alone. This procedure is here applied to ten examples of disordered crystal structures, mostly from a Cambridge Crystallographic Data Center (CCDC) drug subset (Bryant *et al.*, 2019 ▸), where experimental Bragg intensity data were available. Experimentalists often assume that static is different from dynamic disorder, which is misleading. While static and dynamic can be distinguished based on convergence of a disordered fragment to one or two sets of atomic positions in LSQ refinement, taking into account temperature dependence of the atomic displacement parameters (ADPs) and parameter correlation, a more detailed investigation of disorder benefits from the inclusion of computed energies. As will be shown, one can then gain a deeper understanding, and distinguish static and dynamic disorder by combining theory and experiment.

Better refinement of disordered experimental crystal structures is the main result reported here and is of practical relevance. However, procedural improvements reported also have broader implications on how disorder can be understood and categorized through energies. Hence, a general discussion on disorder follows next, before a subsequent description of the methods and results.

## 2. Disorder in the broader context   

### 2.1. Disorder and molecular conformation   

A crystal used to be defined in terms of ideally fulfilling long-range order of a structural arrangement, *e.g.* a molecule in a solid. This definition was altered (IUCr, 1992 ▸) to become ‘any solid having an essentially discrete diffraction diagram’ to ensure that aperiodic or modulated structures, as well as quasicrystals, are also included. When molecules pack to form a crystal with a single molecule in the asymmetric unit (ASU) of the unit cell, usually only one single conformation persists throughout that crystal. When the crystal structure is disordered, there are, in the simplest case, two molecular conformations in the ASU^2^. Since in many such structures there is no perfect long-range order and since X-ray diffraction probes the average structure, the domain-based statistical nature of disorder and the averaging process of diffraction then lead to two structures apparently being present at once. In many such cases, most of the atomic positions are shared between two distinct conformations of a molecule. Thus, in an overlay of both, only the disordered part of a structure differs. In experimental LSQ refinement, overlaying atoms are usually assigned shared positions and displacement parameters. Only atoms further apart than the resolution of the experiment, with separate conformations, usually get assigned separate positional and displacement parameters. From the viewpoint of chemistry, a molecule cannot have two conformations at once because one would have to split elemental particles and therefore models of disordered crystal structures must represent time or space averages of the ASU content. This also holds for molecules on special positions, which cannot be split in half (quarter, *etc.*) but must be present in their entirety. Therefore, a quantum chemical optimization of a disordered structure needs to involve *n* archetype structures.

### 2.2. Disorder, space-group symmetry and diffuse scattering   

Space-group symmetry (Hahn, 2002 ▸; Aroyo, 2016 ▸) describes how applying symmetry operations to the ASU content, often a single molecule, can generate the crystal packing. It has been developed from mathematical group theory to describe idealized non-disordered structures and experimental diffraction patterns. One can think about disorder by starting from the molecular structure or by starting from solid-state symmetry. By removing symmetry from a disordered high-symmetry crystal structure, one can sometimes arrive at a non-disordered subgroup of a particular space group (Aroyo *et al.*, 2006*a* ▸,*b* ▸; Müller, 2013 ▸). If one can then resolve the disorder by LSQ modelling, and if it can be resolved in a lower-symmetry space group, then space-group assignment in the higher-symmetry space group is pseudosymmetrical only. If the disorder persists even with the lowest possible space-group symmetry, then the highest possible symmetry of the disordered ensemble is the correct choice to model diffraction data. One can see from cases where a molecule itself is symmetric, and has a particular point-group symmetry, that it can become difficult to separate molecular and space-group symmetry^3^. Molecular symmetry does obviously need to be considered in the determination of correct space-group symmetry for a disordered molecule. Since an overlay of two different molecular conformations can still fulfil rather high space-group symmetry, I consider it useful to think about disorder starting from a molecule and its environment in the crystal, approximated by a cluster of molecules, to disentangle the complexity of many common disorder phenomena.

The correct highest-symmetry space group (the supergroup) for a disordered experimental structure necessarily violates long-range order because only one conformation can be present in the ASU at the same time. This also applies when molecular conformations in a disordered structure are altered statistically, *i.e.* in a random manner. While an overlay of archetypes fulfils supergroup symmetry, the real physical situation can be an addition of subgroup symmetry plus the symmetry operation that relates the alternating conformations, or just an overlay through translation. It is useful to take space group *P*1 as an example, where there is only translational symmetry. Considering a disordered structure with two molecular conformations that both pack in arrangements of similar shape, charge distribution and energy shows that even in space group *P*1 there can be disorder. In cases where there is a random succession of molecules with either conformation, only a supercell can describe the realistic statistical arrangement of molecules, and a quantum chemical description would need to involve a space (or time) average when the aim is to faithfully reproduce the influence of all possible intermolecular interactions. The situation is different when there are alternating domains where only one conformation is consistently present. Then a molecule-in-cluster approach can provide a very good approximation of that physical situation. Then one can simplify a disordered structure to a sum of conformation A in a cluster of A and conformation B in a cluster of B. I will look at the energetic situation of conformation A in B and B in a cluster of A in subsequent work.

It is often sufficient to consider the disordered ASU with split occupancies of the respective conformer for reproducing experimental diffraction patterns of disordered crystal structures. This description is strictly valid only for Bragg scattering, and I refer to a selection of a considerable amount of work that has been invested in understanding and modelling diffuse scattering (*e.g.* Weber *et al.*, 2001 ▸; Welberry, 2001 ▸, 2004 ▸; Neder & Proffen, 2008 ▸), where a supercell approach is again necessary to reproduce diffuse scattering. While I continue to focus on Bragg scattering for structure elucidation by SC-XRD or neutron diffraction here, one should always be aware of the fact that disorder and diffuse scattering usually appear together.

For some experimental crystal structures, reflections with significantly lower intensity are found in between main reflections. These reflections lead to a larger unit cell. Such supercells are indicative of small differences in molecular conformations and are thus closely related to disorder. Such a situation cannot entirely be separated from thermal diffuse scattering (Willis & Pryor, 1975 ▸), where thermal motion leads to small differences in the atomic positions so that they, strictly speaking, do not fulfil translational symmetry anymore. Both phenomena will not be further investigated or considered here.

## 3. Methods: enriching the toolset to model disorder   

### 3.1. Background on molecule-in-cluster computations   

Next, some background information is provided on molecule-in-cluster computations. Crystallographic ONIOM (Svensson *et al.*, 1996 ▸) MO/MO or QM/MM cluster computations, and those where the same level of theory is used throughout, have in common that molecules in the ASU are expanded to generate a cluster of molecules using space-group symmetry. This is why I jointly call them molecule-in-cluster computations (Dittrich *et al.*, 2020*a* ▸) here. ONIOM cluster computations were first used in SC-XRD to calculate reference values for the internal part of atomic displacements of hydrogen atoms (Whitten & Spackman, 2006 ▸). Later on, it was attempted to compute ADPs of all atoms with the help of empirical scaling (Dittrich *et al.*, 2012 ▸). Other applications were centred on computing geometries (Bjornsson & Bühl, 2012 ▸) and bond distances involving hydrogen atoms (Dittrich *et al.*, 2017 ▸), which can be extended to excited-state conformations of a molecule in the solid (Kamiński *et al.*, 2010 ▸). Even polymorph energy ranking was carried out (Mörschel & Schmidt, 2015 ▸). All these computations have in common that a cluster, which consists of molecules or ions in proximity to a central system, reproduces the crystal environment. The advent of the tight-binding semi-empirical GFN2-xTB (Bannwarth *et al.*, 2019 ▸) approach induces new momentum to cluster computations because of the considerable speed up (Dittrich *et al.*, 2020*a* ▸). What took days (hours) before takes hours (minutes) now, depending on the computer system. Comparably accurate optimized geometries can now be obtained rapidly from conventional starting structures, as provided by independent atom model (IAM) refinements. These are available for a large number of compounds (Taylor & Wood, 2019 ▸). These developments open up several new applications of molecule-in-cluster computations, *e.g.* structure validation (Dittrich *et al.*, 2020*a* ▸), modelling disorder structures with restraints (studied here), complementing or replacing Rietveld refinement (Rietveld, 1969 ▸) in structure solution from powder X-ray diffraction (David & Shankland, 2008 ▸), benchmarking theoretical computations with high-quality experimental diffraction data and investigating co-crystal structures, to name just a few.

### 3.2. Disorder modelling aided by cluster computations   

To improve disorder modelling with the molecule-in-cluster approach, one starts with all *n* possible archetype structures derived from experimental refinement. The preparation entails writing out the *n* disordered parts separately, completing the ASU content with the shared atoms, and computing each of them separately as if they were an ordered structure. Input clusters were generated from a program-specific input file using the program *BAERLAUCH* (Dittrich *et al.*, 2012 ▸) for this purpose, whose symmetry source-code base can be traced back to *LAZY PULVERIX* (Yvon *et al.*, 1977 ▸). *BAERLAUCH* can output a cluster ‘coord’ file in *TURBOMOLE* format (Furche *et al.*, 2014 ▸) that can be processed by *XTB* (Grimme *et al.*, 2017 ▸) [semiempirical extended tight-binding program package *XTB*, https://github.com/grimme-lab/xtb (accessed 20th August 2020)]. These *XTB* molecule-in-cluster computations were then performed in ten repeat cycles to ensure convergence. Only the ASU is optimized, surrounding molecules are kept fixed. After each cycle, the optimization result is evaluated to build a new cluster, using a 3.75 Å distance criterion (of any atom to any atom within the ASU) to select whole molecules surrounding the central ASU molecule(s). Computations cover and optimize all *n* independent idealized disorder conformations. Unit-cell parameters remain fixed at the experimental result.

*BAERLAUCH* subsequently evaluates the optimization result and directly writes *SHELXL* (Sheldrick, 2008 ▸) input with structure-specific restraints. These restraints can also be imported into *olex2.refine* (Bourhis *et al.*, 2015 ▸). It should be pointed out that there are several other sources of restraints. For small-molecule structures, these can be generated from averaged chemical environments considered to be similar in the CSD via the program *MOGUL* (Bruno *et al.*, 2004 ▸), or by using structural input from experiment or theory with the program *DSR* (Kratzert *et al.*, 2015 ▸). An overview of different restraint-generating programs available for macromolecular (ligand) refinement is given in the work of Steiner & Tucker (2017 ▸).

When generating structure- and disorder-specific restraints with *BAERLAUCH*, separate archetype structures need to be recombined to give *n* sets of coordinates for subsequent crystallographic LSQ. Such LSQ refinements need to invoke restraints and constraints to maintain stability and to avoid over-parameterization. *BAERLAUCH* therefore writes out all possible (or a selection of) bond distance (1–2) and angle (1–3) restraints so that each archetype maintains its optimized geometry. To allow for the same atom names and a shared-occupancy parameter in each archetype, these are assigned a separate residue with the RESI command. Commands are written into a separate instruction file. In *SHELXL*, 1–2 and 1–3 restraints are called DFIX and DANG^5^. This means that *n* whole molecules are present in the input files, with duplicate atoms that would otherwise share split sites that become separate sites now. To avoid over-parameterization of such proximate or overlaying atoms, EXYZ and EADP constraints are used. Constraints ensure that the non-disordered parts of a disordered structure, *i.e.* atoms that overlap within the resolution of the experiment, are not assigned separate atomic positional or displacement parameters. A new aspect is that DFIX/DANG restraints allow avoiding of positional EXYZ constraints, since the restraints from the optimization in principle do not make them necessary or even make them redundant. EADP constraints remain necessary, and this is further investigated and discussed below. Since invoking these commands depends on the proximity of ordered or disordered atom pairs, a comparison of archetype structures cannot directly be written by *BAERLAUCH*. An additional program called *SHELXTOOLS* was thus written, which, after reading in the *n* separate archetype structures, compares the distances of atoms pairs provided in the same sequence. It writes out the constraints required; these were generated when atom–atom distances of separate conformers were less than *e.g.* 0.5 Å or a user-specified value. Sometimes different ADPs make physical sense even for proximate atoms and separate ADPs can add more flexibility to a model. Therefore, if so desired, SIMU restraints can be used in place of the EADP constraints, and individual commands required for *SHELXL* are again provided in an additional file. This is possible by evoking a ‘+filename’ command, which tells *SHELXL* to read in commands from the file as if they were contained in the main input file. The different effects of parameterization are tested below.

### 3.3. Compounds studied   

**Figure 1:**
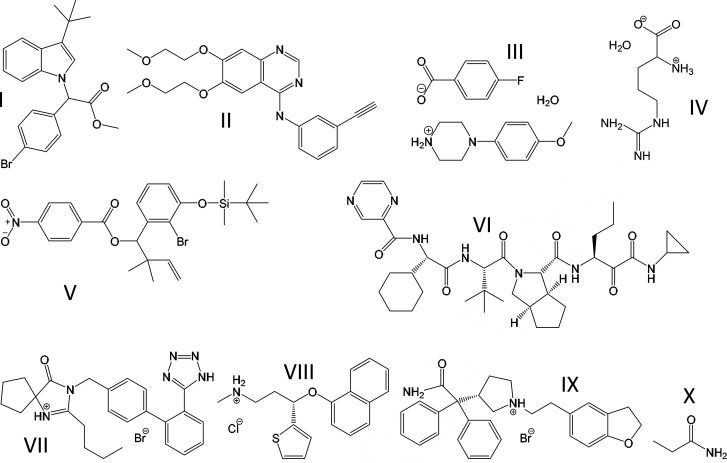
Molecules used for studying disorder in this work, ordered by CSD refcode: **I**, methyl (4-bromo­phenyl)(3-*tert*-butyl-1*H*-indol-1-yl)acetate; **II**, erlotinib; **III**, a cocrystal of MeOPP with 4-fluoro­benzoate and H_2_O; **IV**, DL-arginine monohydrate; **V**, 1-(2-bromo-3-{[*tert*-butyl­(di­methyl)­silyl]­oxy} phenyl)-2,2-di­methyl­but-3-en-1-yl-4-nitro­benzoate; **VI**, telaprevir; **VII**, irbesartan HBr 2H_2_O; **VIII**, duloxetine HCl; **IX**, darifenacin HBr; and **X**, propionamide.

Ten example crystal structures were studied, alphabetically ordered by their CSD refcode. Five disordered compounds from the CCDC drug subset (Bryant *et al.*, 2019 ▸) were initially selected for study. Computations performed in our earlier study (Dittrich *et al.*, 2020*a* ▸) on the drug subset were partly relied upon for these structures. Their diffraction data were deposited alongside publications in the journals of Acta Crystallographica sections C and E. For two further examples, propionamide (Fabbiani *et al.*, 2014 ▸) and DL-arginine monohydrate (Kingsford-Adaboh *et al.*, 2002 ▸) data were collected by the author. Intensity data are usually embedded in deposited CIFs today. This permitted adding three further examples, which were randomly selected based on disorder being reported from a search in the CSD. The datasets used were: **I**, methyl (4-bromo­phenyl)(3-*tert*-butyl-1*H*-indol-1-yl)acetate, refcode CAWWIJ (Arredondo *et al.*, 2017 ▸); **II**, erlotinib, refcode DULKAX (Sridhar *et al.*, 2010 ▸); **III**, 4-(4-meth­oxy­phenyl)­piperazin-1-ium (MeOPP) 4-fluoro­benzoate monohydrate, refcode FOVPOY (Kiran Kumar *et al.*, 2019 ▸); **IV**, DL-arginine monohydrate, refcode FUGXIO01 with intensity data (lattice parameters) from a subsequent charge-density study (Kingsford-Adaboh *et al.*, 2000 ▸, 2002 ▸); **V**, 1-(2-bromo-3-{[*tert*-butyl­(di­methyl)­silyl]­oxy}phenyl)-2,2-di­methyl­but-3-en-1-yl 4-nitro-benzoate, refcode IRUMAL (Ghosh & Kassekert, 2016 ▸); **VI**, telaprevir, refcode LERJID (Gelbrich *et al.*, 2013 ▸); **VII**, irbesartan bromide sesquihydrate, refcode NIQVIT (Wang *et al.*, 2007 ▸); **VIII**, duloxetine hydro­chloride, refcode MUCDUK (Bhadbhade *et al.*, 2009 ▸); **IX**, darifenacin hydro­bromide, refcode SOYMID (Selvanayagam *et al.*, 2009 ▸); and **X**, propionamide, refcode ZZZKAY03 at 100 K (Fabbiani *et al.*, 2014 ▸). For propionamide there is an additional room-temperature structure relevant here, with refcode ZZZKAY05 (Fabbiani *et al.*, 2014 ▸). Lewis formulae of all the compounds are shown in Fig. 1 ▸ and they are discussed next on a case-by-case basis.

## 4. Results and discussion   

### 4.1. Methyl (4-bromo­phenyl)(3-*tert*-butyl-1*H*-indol-1-yl)acetate (refcode CAWWIJ)   

**Figure 2:**
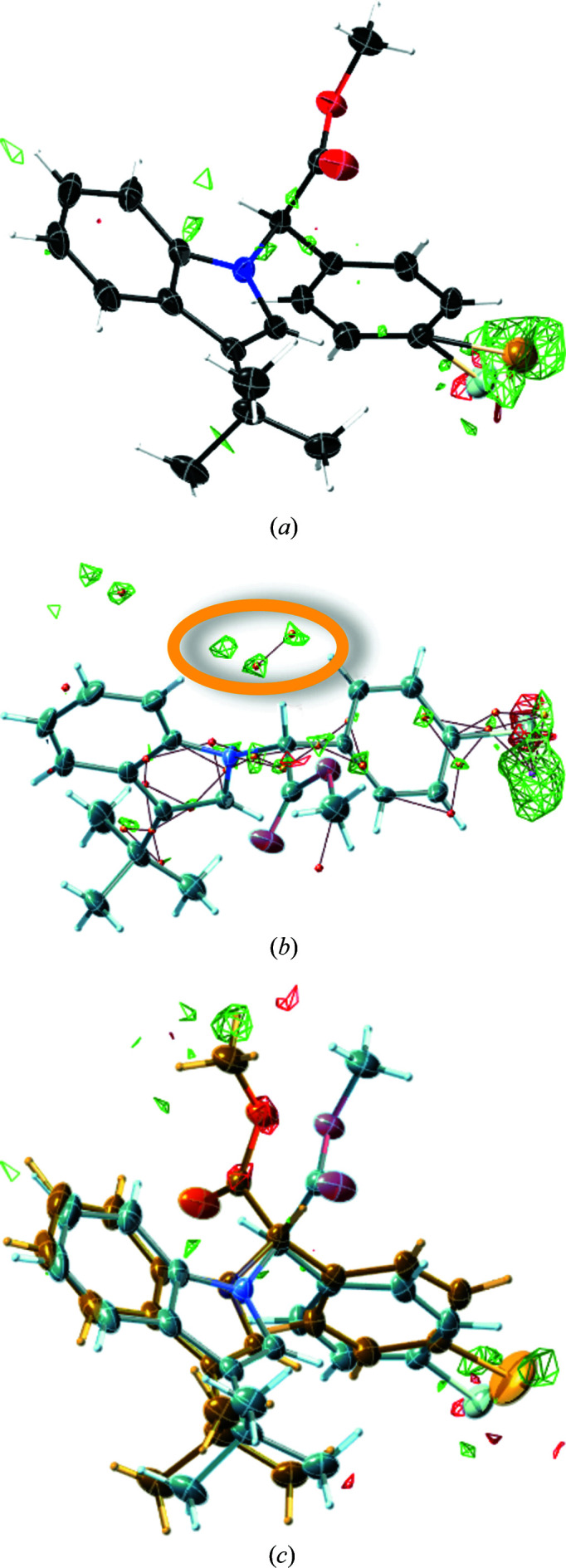
Displacement ellipsoid representations of methyl (4-bromo­phenyl)(3-*tert*-butyl-1*H*-indol-1-yl)acetate, CSD refcode CAWWIJ, generated with *SHELXLE* (Hübschle *et al.*, 2011 ▸). (*a*) The result of the original deposition, with residual-electron-density meshes (0.3 e Å^−3^ throughout) in green. (*b*) The outcome of a restrained (DFIX/DANG) refinement of the major component only, while (*c*) shows the result of a final restrained and additionally constrained (EADP) refinement. For (*b*), additional (highlighted) residual-density peaks for the disordered atoms from the second molecular orientation appear.

The structure of methyl (4-bromo­phenyl)(3-*tert*-butyl-1*H*-indol-1-yl)acetate, refcode CAWWIJ, is our starting point. Here the only initial indication for disorder is a second site of a bromine atom in the residual electron density [Fig. 2 ▸(*a*)] and the structure seems to be that of an enantiopure material. Owing to the large number of electrons in bromine, even the small occupancy of 3% is clearly visible. Other atomic sites of a second disorder component initially do not have a signal above 0.3 e Å^−3^. Molecule-in-cluster optimization of the major component with 97% occupancy, and using it for restraining the model in subsequent refinement of the major component only, leads to a pronounced residual-electron-density peak for the second bromine atom. In addition, additional peaks appear [Fig. 2 ▸(*b*)] for the non-overlapping atoms from the second disorder conformation or archetype structure, mainly involving its ester functionality.

Including archetype-specific restraints of the main disorder component thus increases the signal for the un-modelled disorder. This in turn provides initial coordinates for computing the second archetype of different handedness. Restraints derived from the second computation then permit describing this second archetype in the model to give a better result in terms of information content. *R*(*F*) was reported to be 4.47% in the literature with the model already including a second site of the bromine atom, albeit with dubious molecular geometry. Including restraints from the second archetype structure in the refinement (using the same OMIT and SHEL commands as in the deposited CIF) reduces the *R* factor to 4.37%, despite the minor 3% occupancy of the second configuration. While modelling the disorder from the perspective of figures of merit hardly merits the effort, the disorder is directly related to the ability to determine the enantiopurity of the compound. Since this is important information for synthetic work (Arredondo *et al.*, 2017 ▸), being able to model the disorder better has the important implication that the compound is not enantiopure. Energy differences between the two different configurations are not expected and this is confirmed from our computations (see Table 2).

### 4.2. Erlotinib (refcode DULKAX)   

**Figure 3:**
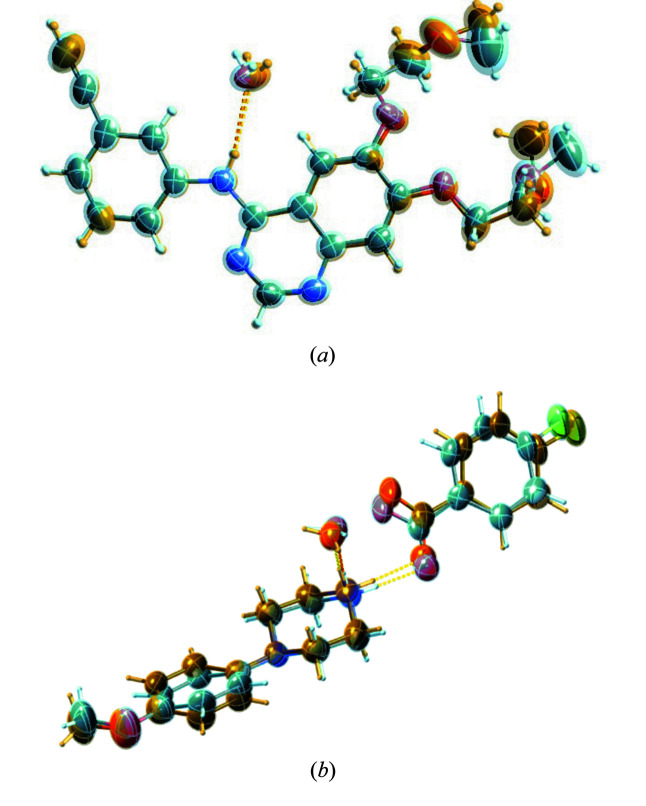
Displacement ellipsoid representations of the structure of erlotinib [CSD refcode DULKAX, (*a*)], highlighting the almost perfectly overlaying atoms and the disordered parts of the different conformations of the molecules in the disordered structure, and the cocrystal structure of 4-(4-meth­oxy­phenyl)­piperazin-1-ium MeOPP 4-fluoro­benzoate monohydrate [CSD refcode FOVPOY, (*b*)]. The illustrations were generated with *SHELXLE* (Hübschle *et al.*, 2011 ▸) using the restrained (DFIX/DANG) and additionally constrained (EADP) refinements I recommend.

For erlotinib I initiated the molecule-in-cluster computations with the two conformers present in the erlotinib structural model [Fig. 3 ▸(*a*)]. These were first modelled as different ‘PARTs’ in *SHELXL2018* (Sheldrick, 2015 ▸), written out as separate archetype structures and then energy minimized at the GFN2-xTB level of theory with *XTB*. Extracting these two conformers could be considered as generating ‘different polymorphs’ from the same crystal structure, were they exclusively present. This procedure was subsequently followed throughout. Erlotinib (refcode DULKAX) in *P*1 shows disorder in both 2-meth­oxy­eth­oxy side chains. Both archetype structures overlay nicely before and after refinement with restraints [Fig. 3 ▸(*a*)].

It should also be emphasized how well the bond-distances and bond-angle restraints from GFN2-xTB molecule-in-cluster computations fit the experimental data for disordered molecules. This was observed earlier for high-quality low-temperature data only (Dittrich *et al.*, 2020*a* ▸). The use of molecule-specific restraints is conceptually close to rigid-body refinement, since model flexibility is severely constrained: depending on the restraint s.u.’s, here 0.005 throughout, only changing torsion angles, shifting the centre of mass of the molecule and adjusting the atomic displacements are easily possible, since a value of 0.005 is small. When the restraint s.u. is even closer to zero, restraints effectively become constraints, the difference only being program implementation.

Concerning refinement strategy, the LSQ refinement *R* factor quickly drops to 3.3% when only applying DFIX/DANG restraints. The results reported in the literature were 4.3%, where optimized structure and restraints were not used. However, this comparison would be ‘unfair’, since ADPs are independently refined for atoms in proximity, increasing the number of parameters. Constraining with EADP commands the ADPs of those atoms, which are closer than the resolution of the experiment (here 0.5 Å was chosen), still gives significantly better results, *R*(*F*) = 3.9%, than the refinements reported in the literature. The same deposited X-ray diffraction data were used throughout. The best strategy of how to restrain and constrain parameters will be discussed below after an overview of all the structures evaluated in a similar manner in Table 1 ▸, shown above. The GFN2-xTB energy difference between the two archetype structures (normalized to one ASU content throughout) is only 1.0 kJ mol^−1^.

### 4.3. MeOPP 4-fluoro­benzoate monohydrate (refcode FOVPOY)   

Studying the structure with refcode FOVPOY [Fig. 3 ▸(*b*)], which is part of a group of disordered isomorphous structures of 4-(4-meth­oxy­phenyl)­piperazin-1-ium salts (Kiran Kumar *et al.*, 2019 ▸), raises questions, since from a quantum chemical point of view its structure, although modelled as such, might not directly be considered as disordered. While both archetypes A and B, as generated from the deposited experimental structure, converge to different minima with an energy difference of 4.03 kJ mol^−1^, and while the optimized ASU coordinates are not identical for A and B, there is no obvious conformational difference in the optimized co-crystal structure archetypes. Qualitative equivalence of their conformations can be shown by calculating the root-mean-square Cartesian displacement (van de Streek & Neumann, 2010 ▸) between ASU coordinates of A and B, which is 0.17 Å. What causes the disorder in this example is initially unclear. A closer look reveals the cause of disorder to be the repulsion between the piperazin-1-ium and the meth­oxy­phenyl hydrogen atoms.

The structure analysis of the room-temperature diffraction data of FOVPOY was published with a comparably high *R* factor of 6.6% and the authors omitted diffraction data above 52° in Θ in their refinement. Cutting the data further to 0.84 Å resolution in refinement reduces *R*(*F*) considerably. This means that high-resolution data are rather noisy. Refining the published disorder model at 0.84 Å does not give an improvement of *R*(*F*) and there is no signal of residual electron density at this resolution that could be assigned to discrete atoms, which supports an alternative conformation. Disorder only barely becomes visible in the meth­oxy­phenyl ring when including the high-order diffraction data to 52° in Θ. Experimental data alone do not give an unambiguous answer for FOVPOY. Only when cutting the data to 0.84 Å and using DFIX/DANG restraints and EDAP constraints does the modelling of disorder show the expected benefits (see Table 1 ▸), but this requires having modelled the second archetype in the first place.

What is the lesson learnt from this example? Is it that quantum chemistry cannot always tell whether there is disorder or not? Or that when quantum chemistry predicts it, it’s real? Another aspect might lead to a conclusion: vibrational motion of molecule(s) in a shallow energy landscape often leads to large-amplitude vibrations. Such vibrations can be modelled by introducing disorder in refinement, which cannot be described well with anisotropic ADPs using one site per atom. Modelling large-amplitude vibrations with ‘banana shaped’ ADPs [as possible with the program *CRYSTALS* (Betteridge *et al.*, 2003 ▸)] rather than split sites could hide the physical reality of disorder, when it is confirmed by computations. The authors did well to recognize it for FOVPOY.

### 4.4. DL-arginine monohydrate (refcode FUGXIO01)   

**Figure 4:**
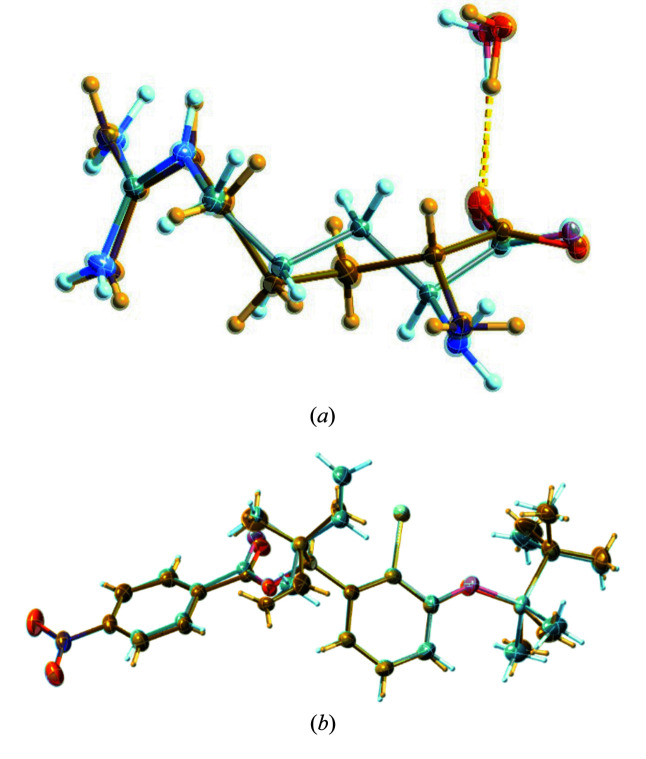
Displacement ellipsoid representations of the molecular structures of DL-arginine monohydrate [CSD refcode FUGXIO01, (*a*)] and 1-(2-bromo-3-{[*tert*-butyl­(di­methyl)­silyl]­oxy} phenyl)-2,2-di­methyl­but-3-en-1-yl 4-nitro­benzoate [CSD refcode IRUMAL, (*b*)], highlighting the disordered parts of the different conformations of the molecules in the disordered structures. The illustrations were generated with *SHELXLE* (Hübschle *et al.*, 2011 ▸) using the restrained (DFIX/DANG) and additionally constrained (EADP) refinements I recommend.

For DL-arginine monohydrate, refcode FUGXIO01, data of unusually high resolution (sin θ/λ = 1.4 Å^−1^) were available. Disorder was initially overlooked (Kingsford-Adaboh *et al.*, 2000 ▸, 2002 ▸) and only observed using difference Fourier maps, available *e.g.* in *SHELXLE* (Hübschle *et al.*, 2011 ▸). Disorder was then reported in a study on modelling disorder with aspherical scattering factors (Dittrich *et al.*, 2016 ▸), where it was found that a minor component is present for only <3%. Requirement (iii) that competing hydrogen-bonding patterns should be of similar strength for disorder to be present in a crystal structure (Dittrich *et al.*, 2018 ▸) is confirmed. A depiction of the two conformers can be found in Fig. 4 ▸(*a*). Combined restrained/constrained refinement of disorder gives an *R*(*F*) or 3.93%, compared with 3.87% with one ADP becoming non-positive definite without EADP constraints. Unlike for all the other molecules studied here, aspherical atom refinements were already performed on this structure. Therefore, our current refinements do not match the multipole/invariom refinements reported earlier, which leads to the best *R* factors [3.0% for free multipole refinement without considering disorder, 2.9% for invariom refinement (Dittrich *et al.*, 2013 ▸) including disorder in the model]. This is owing to additionally modelling the non-spherical electron density in these latter refinements, which for multipole refinement requires high-resolution data. It was emphasized that high resolution is beneficial for structural work in general (Sanjuan-Szklarz *et al.*, 2016 ▸), and it certainly helps modelling disordered structures as well. In addition, the high-resolution data confirm that the 1–2 and 1–3 restraints nicely fit the experimental data even at such high resolutions within small restraint s.u.’s

Interestingly for DL-arginine monohydrate, the energy difference of the two archetype structures normalized to one ASU is apparently higher than *RT*, with 16.3 kJ mol^−1^ from GFN2-xTB computations, but still rather close. To increase the accuracy of this result, I additionally performed two-layer ONIOM (Svensson *et al.*, 1996 ▸) optimizations with the method/basis set combination B3LYP/6-31G(d,p):B3LYP/3-21G with GD3 dispersion correction (Grimme *et al.*, 2010 ▸), starting from the invariom geometry from 2016 on 16 (molecule + water) clusters. Here the difference of the high-layer energies for each of the archetype structures is, with 2.57 kJ mol^−1^, very close to *RT* (2.48 kJ mol^−1^, *T* = 298 K).

### 4.5. 1-(2-bromo-3-{[*tert*-butyl­(di­methyl)­silyl]­oxy}phenyl)-2,2-di­methyl­but-3-en-1-yl 4-nitro­benzoate (refcode IRUMAL)   

For the structure with refcode IRUMAL [Fig. 4 ▸(*b*)], refinement *R* values using DFIX/DANG with and without EADP are broadly similar to the originally published result. Improved *R* factors can only be achieved when loosening the restraint s.u’s. One can therefore wonder whether the proposed method really adds value here. To answer this concern, it is argued that modelling disorder is often a time-consuming activity that requires manual work and expertise, and that the computations provide a clear path to automate such activity. Following this path also leads to consistent results without a need for user-dependent compromises in parameterization. So even when the *R* factor does not merit the effort, the reduction in manual-modelling effort might. Moreover, the computation of IRUMAL archetypes provides a valuable result and shows that the energy is again within 2.26 kJ mol^−1^ *i.e.* within *RT*.

### 4.6. Telaprevir (refcode LERJID)   

**Figure 5:**
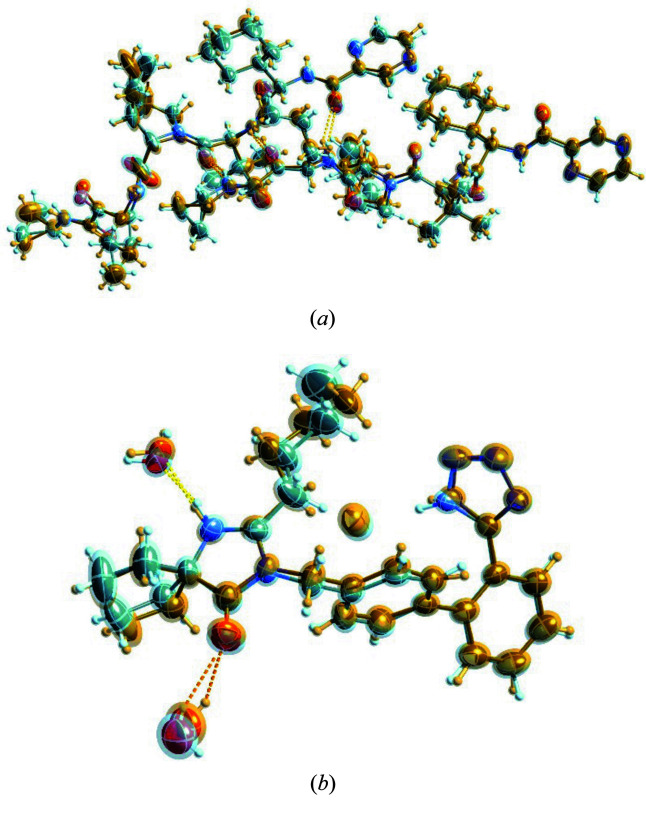
Displacement ellipsoid representations of the structures of telaprevir [CSD refcode LERJID, (*a*)] and irbesartan bromide sesquihydrate [CSD refcode NIQVIT, (*b*)]. For molecules the size of LERJID, finding the overlaying atoms and assigning EADP constraints can be facilitated by suitable software. The illustrations were generated with *SHELXLE* (Hübschle *et al.*, 2011 ▸) using the restrained (DFIX/DANG) and constrained (EADP) refinements I recommend.

The molecules **V** to **IX** are rather large, at least compared with propionamide **X**, with telaprevir (**VI**, refcode LERJID) being the largest one. Its archetype structures include side-chain conformations that differ considerably [Fig. 5 ▸(*a*)].

For disorder, size matters. It would be interesting to correlate molecular size to the frequency of occurrence of disorder in a structure. I suspect that conformations of similar shape, charge density and energy become more probable with increasing molecular size. Energies of the archetype structures from LERJID, normalized to one ASU, are again – similar to FUGXIO01 and DULKAX – very close, with an energy difference of 0.8 kJ mol^−1^. Archetype conformers might thus easily interconvert in solution, but not in the solid state, when they pack in the same crystal structure, depending on energy barriers. A new requirement (iv) that the energy of archetypes should be very close should, therefore, be added to the abovementioned criteria for disorder to occur (Dittrich *et al.*, 2018 ▸). However, enthalpy does not explain everything and will be just one side of the coin, the other side being a molecule’s vibrational behaviour. Concerning LSQ refinement of LERJID, free restrained refinement of disorder gives an *R*(*F*) of 3.1% with several non-positive definite ADPs, compared with 3.7% with additional EADP constraints. The literature result is 4.7%.

### 4.7. Irbesartan bromide sesquihydrate (refcode NIQVIT)   

For the hydrate of the bromide salt of irbesartan [refcode NIQVIT, Fig. 5 ▸(*b*)], the same refinement strategies were followed as before: (1) free LSQ refinement with all possible DFIX/DANG restraints with a restraint s.u. of 0.005 and (2) additionally constraining the ADPs of proximate atoms within 0.5 Å. The *R* factor for (1) was 3.8% but many of the overlaying atoms became non-positive definite and had to be constrained with the XNPD command in *SHELXL*. For refinement strategy (2), the *R* factor increased to 4.2% but stayed considerably below the result (4.9%) reported in the literature. Another alternative refinement choice would be to refine the ADPs with restraints rather than constraints for disordered atoms in proximity. Here using SIMU restraints with a s.u. of 0.02 in *SHELXL* leads to an *R* factor of 3.8% but the orientation and shape of the ADPs remains dubious. Reducing the s.u. to 0.005 then gives more physically looking ADPs and an *R* factor of 4.1%. Here, different crystallographers can and will make different choices; I recommend using EADP constraints, since this is a simple procedure and leads to the most physically meaningful ADPs.

Like for the three molecules considered before, I again observed similarity in the ASU energies of the archetype structures, with an energy difference of only 0.2 kJ mol^−1^ at the GFN2-xTB level of theory. The energies again do not seem to exceed a particular threshold, namely *RT* at room temperature. Assuming that crystals are usually grown at ambient conditions, this threshold indicates what energy is available for conformational flexibility of molecules crystallizing into disordered structures.

### 4.8. Duloxetine hydro­chloride (refcode MUCDUK)   

Free restrained refinement of duloxetine HCl (refcode MUCDUK), with two full archetype molecules present, leads to an *R*(*F*) of 4.2%, but five non-positive ADPs. For the literature result of *R*(*F*) of 4.4%, the authors already used 110 restraints and 237 parameters. GFN2-xTB restrained/constrained refinement gives an *R* factor slightly below 4.4%, less impressive than but consistent with the earlier cases.

**Figure 6:**
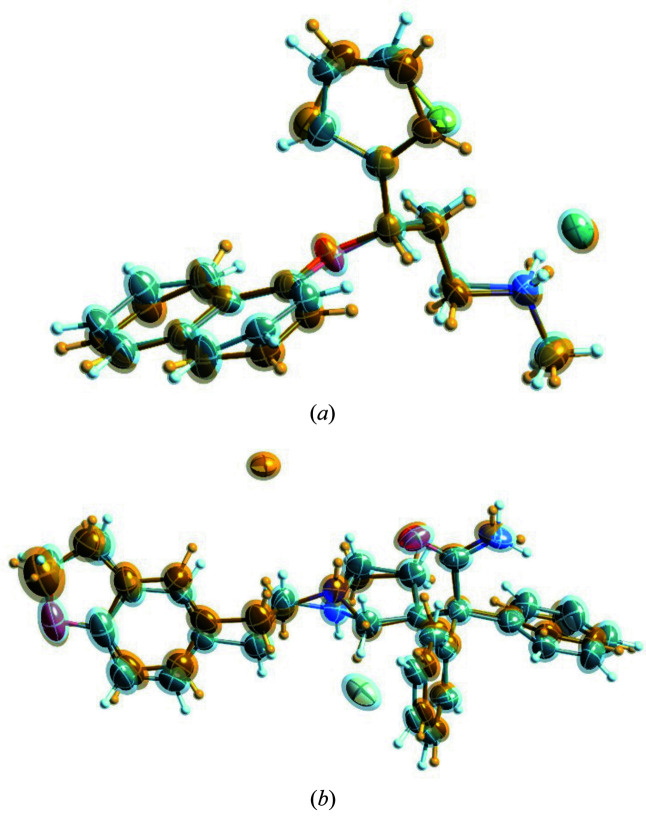
Displacement ellipsoid representations of the structures of duolexetine hydro­chloride [CSD refcode MUCDUK, (*a*)] and darifenacin hydro­bromide [CSD refcode SOYMID, (*b*)] from restrained/constrained refinements. In these two cases, only the overall shape of the molecules superpose well. Disordered parts of the different conformations of the molecules are highlighted again. The illustrations were generated with *SHELXLE* (Hübschle *et al.*, 2011 ▸).

The energy difference between the conformers is 1.1 kJ mol^−1^. It can be seen from Fig. 6 ▸(*a*) that the overlay of atoms is not as strong as for the earlier structures and that the overall shape of both archetype conformations [shape requirement (ii) mentioned in the introduction] fits well.

Unlike in conventional disorder refinements, an overall occupancy free variable was assigned to the entire archetype molecule(s) in the refinements reported. Usually only the disordered atoms are assigned an occupancy parameter. Another technical detail is that all bond distances to hydrogen atoms were shrunk by 13.5% for generating restraints when going from theory to experiment in all cases.

### 4.9. Darifenacin hydro­bromide (refcode SOYMID)   

Studying darifenacin HBr (SOYMID) further confirms results seen so far: *R* factors for restrained refinement lead to one non-positive definite ADP but several unphysical looking ADPs, indicating over-parameterization, and the best *R* factor (4.2%). Like for all earlier cases, LSQ refinement remains robust and stable owing to the restraints. This is a valuable result in itself because of the time saved to test (failing) modelling strategies during refinement of disordered structures. To obtain physically meaningful ADPs, one has to constrain the ADPs of proximate atoms. This leads to an increase in the *R* factor to 4.4%, which is still significantly better than the result reported in the literature (5.4%) using the same intensity data. Like already seen for MUCDUK, for SOYMID the atoms overlay less well than for NIQVIT and DULKAX [Fig. 3 ▸(*a*)], so the distance threshold of when to use constraints increases user choice. The mixed restrained/constrained refinement is also most convincing for SOYMID. It is interesting that the disorder in SOYMID is driven by the position of the hydrogen atom at the central nitro­gen atom. Either it is pointing upwards or downwards, which affects the most suitable (but symmetry equivalent) position to place the bromide ions in vicinity to this hydrogen for a well balanced cluster.

SOYMID archetype energy differences are, with 0.9 kJ mol^−1^, again smaller than *RT*, with *T* at 293 K. Since *BAERLAUCH* cannot handle atoms on special positions yet, it was assumed that the second water molecule was fully occupied in the computations. Another interesting detail also emerges. Unlike the situation for modelling IAM residual density with aspherical scattering factors, where light atom structures fare better, modelling disorder with archetype-specific restraints becomes more important the more a structure includes heavier elements, exemplified by the bromide salts NIQVIT and SOYMID. This is because of the smaller relative ratio of valence electrons (Stevens & Coppens, 1976 ▸; Dittrich *et al.*, 2006 ▸), as captured with the diffraction-precision-index value (Blow, 2002 ▸) when heavier atoms are present, since core scattering affects the entire resolution range of a dataset, in contrast to scattering from more diffuse valence electron density in bonds, lone pairs and *d* orbitals.

### 4.10. Propionamide (refcode ZZZKAY03)   

**Figure 7:**
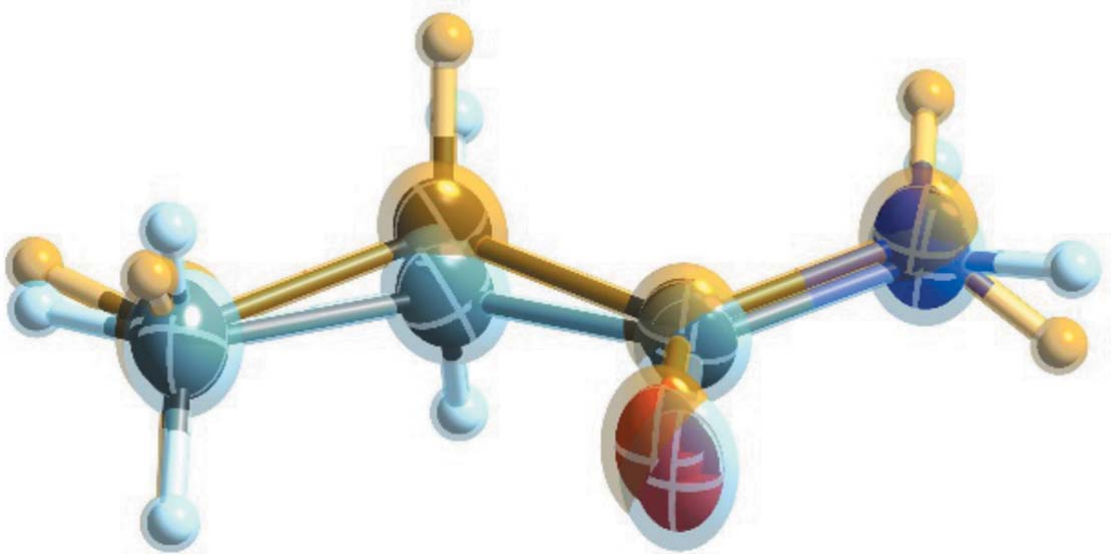
Displacement ellipsoid representations of the molecular structure of propionamide, CSD refcode ZZZKAY03, from restrained/constrained refinement. The illustration was generated with *SHELXLE* (Hübschle *et al.*, 2011 ▸).

For propionamide **X**, datasets at two temperatures were measured. Here, the disorder can be resolved at a temperature of 100 K (Fig. 7 ▸) and the two archetype structures optimize to distinct nonplanar local minima. At room temperature, modelling two split sites does not improve the modelling (Fabbiani *et al.*, 2014 ▸) and an overall weaker fit to the data of the conformational average with single positions was observed. This can be explained by moving away from an ideal crystal with more perfect translational symmetry at room temperature. Therefore, this is a case of dynamic disorder, where the molecule overcomes the energy barrier when the thermal energy available to the system permits it.

Like for the other structures, I obtained similar energies for the two archetype structures of propionamide. The molecule is non-planar in the crystal, the two conformers seen at 100 K are owing to the two possible (up/down) ways to deviate from planarity, and their archetype energy is rather close. Computations show that, with the lattice parameter from the 100 K measurement, their GFN2-xTB energy difference is 0.6 kJ mol^−1^. Since the energy barrier is also in a range of *RT* (see discussion below), increasing the temperature (and space available for each molecule) allows conformational changes between the two archetypes at room temperature. The crystal packing remains unchanged upon increasing temperature, owing to the conformations between the two archetype structures being alike and since the amide–amide interactions ‘hold the crystal together’. The small difference of the energies of the archetype structures, together with an energy barrier below *RT*, explains the dynamic behaviour seen in propionamide at room temperature from the structure at 100 K.

The restraints extracted from propionamide fit the data slightly less well than for all the other structures, since the amide hydrogen atoms become non-planar in the GFN2-xTB computations. The semi-empirical approach taken seems to lack basis functions to describe such groups with the required precision, which is also seen for the guanidine group in DL-arginine [Fig. 3 ▸(*a*)], where the results were also less accurate than from MO/MO ONIOM optimization. One could, therefore, omit the restraints involving the affected hydrogen atoms in propionamide, but the effect on the *R*(*F*) is small. The *R*(*F*) for free refinement is 4.2% with two ADPs becoming non-positive definite, for constrained and restrained refinement it is 4.3%. The literature result is 4.9%.

Since the intermolecular interactions of the amide group stay the same for both archetypes, an approximate energy barrier for propionamide can be obtained by focusing on the molecule only. A computation involving an all-planar conformation was generated with *Avogadro* (Hanwell *et al.*, 2012 ▸), with starting values from a universal force field optimization (Rappé *et al.*, 1992 ▸). B3LYP/6-31G(d,p) optimization with *GAUSSIAN09* (Frisch *et al.*, 2013 ▸) then provided values for the planar higher-energy conformation. Starting optimization from either of the two experimental conformations affords the minimal energy conformation in the solid state, and their difference provides an estimate for the energy barrier of 0.2 kJ mol^−1^ at this level of theory. I am confident that more sophisticated computations would confirm that the energy barrier is below *RT* at room temperature.

## 5. Overall discussion, best refinement procedure and occupancies/energies   

Rather than discussing disorder in terms of static or dynamic, examples provided show that it is more helpful to think of it in terms of enthalpy or Gibbs free energy. This leads to a requirement (iv) that I would like to add to the earlier requirements mentioned in the introduction of this article, namely an energy requirement: for disorder to occur, different conformations of similar energy need to be available during crystallization, depending on their Boltzmann population, to become present statistically or in larger domains in the solid. Static and dynamic disorder can then further be distinguished through an energy barrier that may permit conversion between conformers of similar energy. If a barrier can be overcome at ambient conditions, like for propionamide, within an energy window provided by *k*_B_ *N*_A_ *T*, then disorder can be classified as dynamic. Dynamic disorder can then not only disappear with increasing temperature (or pressure) but can also be present to a different degree and frozen in (Dittrich *et al.*, 2020*b* ▸). It remains static, when conformers of similar energy can crystallize in the same packing during the crystallization process, but cannot interconvert without re-dissolution, with the energetic barrier to be overcome from the crystal packing being too high.

These findings also have implications on how to access polymorphs: similarity of the energy (within *RT*) is observed for many polymorphs as well as for disordered structures. A unique requirement for polymorphism to occur then is the control of the conditions like solvent, temperature and pressure during synthesis and crystallization that give just one (or groups of) molecular conformation(s) in the crystallization process. Finally, yet importantly, the distinction into relative energy differences and energy barriers could also explain the phenomenon of disappearing polymorphism (Dunitz & Bernstein, 1995 ▸; Bučar *et al.*, 2015 ▸): polymorphs of similar energy can disappear when a conversion energy barrier can be overcome, given particular conditions.

Now to the question of which is the best refinement model. Table 1 ▸ summarizes *R* factors of the refinements and models discussed above. One can see that the over-parameterized ‘free’ refinement model generally yields the best figures of merit from the models discussed. However, ADPs often become non-positive definite in these refinements and free (over-parameterized) refinement cannot be recommended for use (Hamilton, 1965 ▸). Crystallographers are taught to minimize the number of necessary parameters. This ensures a good data-to-parameter ratio and minimizes parameter correlation, leading to physically meaningful results. Free refinement with doubling atomic sites *x*, *y* and *z* and invoking six additional ADP parameters per atom is clearly not the right refinement strategy. While the restraints keep those refinements stable and capture the small deviations in positions of nearly (but not perfectly) overlaying atoms, *e.g.* as shown in Fig. 6 ▸ for MUCDUK and SOYMID, it is better to constrain the ADPs for overlaying atoms in proximity.

More conservative crystallographers, who might be less enthusiastic about combining theory and experiment (which they already do when using a scattering factor for their atoms), might ask why go back to refinement after the molecule-in-cluster optimization at all. Energies from the computations provided the insight into energy-similarity requirement (iv) and can provide a distinction between static and dynamic, but is there really a need to then go back to the experimental data with restraints?

The strongest argument in favour is the better refinement outcome achieved by capturing the differences of atoms in proximity. Adding EXYZ constraints would, therefore, unnecessarily limit model flexibility. The reason for combining the restraints from theory for disorder refinement is to get the best possible experimental outcome, consistent with theory. Restraints do not only permit implicit cross validation (Dittrich *et al.*, 2020*a* ▸) but also give lower *R* factors. The drop in *R*(*F*) is proportional to the reduction in parameter standard deviations, meaning that the physical significance of the experimental result increases. At the same time s.u.’s of the restraints extract information contained in the experimental diffraction data. While the lowest *R*(*F*) is not the only factor to look at, a lower *R*(*F*) for a similar number of parameters really translates into more accurate and precise structural information. It can be expected that the number of warnings for unusual conformations in *MOGUL* structural checks will be reduced this way. The best refinement model, and one consistent with quantum chemical energy minima, bond distances and angles, is therefore the combined DFIX/DANG restrained IAM refinement, which could further be improved with aspherical scattering factors (Lübben *et al.*, 2019 ▸, and references therein].

Energy differences between archetypes have already been mentioned and discussed. A related aspect is that it is possible to correlate experimental occupancies from SC-XRD to the GFN2-XTB energies. While the energy accuracy is not as high as in the more sophisticated dispersion-corrected density-functional-theory computation carried out for FUGXIO01, I see the expected correlation (Table 2 ▸) that the higher occupancy usually corresponds to the lower-energy archetype, except for DULKAX, MUCDUK and ZZZKAY03, where the occupancies are 50/50 or close to it. A satisfactory finding given the accuracy of the computations.

Concerning the term ‘archetype’, introducing a new word might be useful, since ‘archetype’ does not only permit a better distinction of different disorder components in terms of enthalpy but also helps to explain disappearing disorder and can cover the distinction between individual polymorphs and disorder (Dittrich *et al.*, 2020*b* ▸) through Gibbs free energy.

As a final comment on the CIF format: this file format does not currently distinguish both PART and RESI information from *SHELXL* files. PART information is contained in ‘_atom_site_disorder_assembly’ and ‘_atom_site_disorder_group’ entries. However, which disordered atoms belong to which molecule(s) is not captured. When archetype structures are combined to one disordered structure and are deposited together, some information might get lost. This might also be part of the reason why programs like *MERCURY* (Macrae *et al.*, 2020 ▸) are not currently able to display disordered structures (bonds are missing) as well as *SHELXLE* (Hübschle *et al.*, 2011 ▸). It might thus be useful to add RESI information entries to the CIF standard.

## 6. Conclusions and outlook   

The focus of this work was to facilitate the modelling of disordered crystal structures through the generation of molecule-specific restraints from fast GFN2-xTB quantum chemical molecule-in-cluster computations. This way the quality of the modelling improves and the information content that a disordered structure can provide increases. The human time spent on disorder modelling can be reduced by letting computers work for us. This only requires a quantum chemically plausible archetype starting structure with the roughly correct conformations to initiate a computation, whose restraints then complete disorder refinement rapidly. Improved modelling of disordered crystal structures can, therefore, be an improvement in quality and efficiency, since resolving disorder using only experimental input can be a time-consuming activity that requires chemical intuition and experience. The tool *BAERLAUCH* facilitates computational and post-analysis work, *e.g.* preparing structural input for matching disordered experimental structure archetypes for comparison with CSP landscapes.

Adding energetic information to disordered structures, even at the comparably inaccurate GFN2-xTB level, then better classifies disorder in crystallography. First, it is found that archetype structures, when crystallization takes place at ambient conditions, are very similar in energy, namely around *RT*, the energy available to a molecule at ambient (*i.e.* crystallization) conditions. Further computations on structure-specific energy barriers then put into context categories of static or dynamic disorder. Whether disorder is static or dynamic, disorder is a consequence of how a system responds to the energy available to it at a particular temperature and pressure. When an energy barrier between distinct conformations is smaller than *RT*, the disorder is likely to remain frozen in. Only when it is possible to overcome the barrier above a certain temperature does the disorder become dynamic. We expect polymorphism when differing conformations can be accessed through synthesis, solvent or crystallization conditions. In short, similar energies in the same packing lead to disorder, similar energies in different packings lead to polymorphism. Furthermore, distinguishing static and dynamic disorder by combining theory and experiment in quantum crystallography can be achieved from a single low-temperature experiment, predicting behaviour at higher temperatures. This can guide efforts to perform further multi-temperature experiments, *e.g.* predicting when the energy barrier can be overcome. To reduce the likelihood of disorder it can be recommended to crystallize at lower temperatures, which is possible for *e.g.* vapour-diffusion experiments, to reduce *RT*. As an outlook, I am currently investigating further thermodynamic classification of disorder concerning enthalpy and entropy, and this will be discussed in a subsequent article. Alongside these efforts, the accuracy of the computations will need to be improved and benchmarked, as the example of DL-arginine monohydrate shows.

## Figures and Tables

**Table 1:** A summary of the three refinement results reported in the main text For refcode FOVPOY, the data (in brackets) were cut to a resolution of 0.84 Å.

No.	CCDC refcode	*R*(*F*) (%) from free refinement	*R*(*F*) (%) from restrained/constrained refinement	*R*(*F*) (%) reported in the literature	No. of parameters/restraints for free refinement	No. of parameters/restraints for constrained plus restrained refinement
**I**	CAWWIJ	4.1	4.4	4.5	628/268	469/268
**II**	DULKAX	3.3	3.9	4.3	743/292	578/292
**III**	FOVPOY	6.0 (5.3)	6.7 (5.8)	6.6	636/256	465/256
**IV**	FUGXIO01	3.86	3.93	2.9†	364/140	278/140
**V**	IRUMAL	3.1	3.1	3.0	853/365	657/365
**VI**	LERJID	3.1	3.7	4.7	2614/1236	1958/1236
**VII**	NIQVIT	3.8	4.2	4.9	897/376	672/376
**VIII**	MUCDUK	4.2	4.4	4.4	547/499	442/499
**IX**	SOYMID	4.2	4.4	5.4	844/772	687/772
**X**	ZZZKAY03	4.0	4.3	4.9	148/58	113/58

† For refcode FUGXIO01, the *R*(*F*) results were obtained by aspherical atom refinement, which is not further taken into account here.

**Table 2:** A summary of energies and their relation to occupancies

No.	CCDC refcode	Occupancy of major component	Occupancy of minor component	Energy of major component, minor component set to ± 0.0 (kJ mol^−1^)
**I**	CAWWIJ	0.97	0.03	−0.16
**II**	DULKAX	0.52	0.48	−1.0 (per molecule)
**III**	FOVPOY	0.60	0.40	−4.03 (both converge to similar conformation)
**IV**	FUGXIO01	0.97	0.03	−16.3 (−2.57 from ONIOM)
**V**	IRUMAL	0.80	0.20	−0.54
**VI**	LERJID	0.71	0.29	−0.82
**VII**	NIQVIT	0.66	0.34	−0.23
**VIII**	MUCDUK	0.58	0.42	+1.08
**IX**	SOYMID	0.66	0.34	−0.91
**X**	ZZZKAY03	0.50	0.50	+0.60

## Footnotes

1 An ‘archetype’ is not just an idealized non-disordered structure extracted from a disordered structure. It can include polymorphs and explain disappearing disorder (Dittrich *et al.*, 2020*b* ▸) by linking structure and energy.

2 Substitutional disorder, where different elements occupy the same atomic site, as is common in inorganic chemistry but not limited to inorganic compounds, will not be considered here. An intriguing example in this context is bond-stretch isomerism, which is related to substitutional disorder (Parkin, 1993 ▸).

3 Hydrogen atoms have already been discussed to sometimes be symmetry disordered in optimized crystal structures (van der Streek & Neumann, 2010 ▸). This can be generalized to other atoms involved in disorder.

5 DFIX and DANG restraints are not distinguishable from the program-implementation point of view. The only difference is the default s.u. *SHELXL* assigns to them (0.02 for DFIX and 0.04 for DANG).
